# Impact of recalcification on procoagulant collagen-and-thrombin–activated (COAT) platelet generation in platelet-rich plasma samples with low platelet counts

**DOI:** 10.1016/j.rpth.2026.103372

**Published:** 2026-01-29

**Authors:** Lydia Hayenga, Lucas Veuthey, Manuel Krüsi, Kamand Haeri, Debora Bertaggia Calderara, Lorenzo Alberio, Alessandro Aliotta

**Affiliations:** Division of Hematology and Central Hematology Laboratory, Lausanne University Hospital (CHUV) and University of Lausanne (UNIL), Lausanne, Switzerland

**Keywords:** annexin V, pre-analytic, procoagulant COAT platelet, recalcification, thrombocytopenia

## Abstract

**Background:**

Assessment of platelet procoagulant function by flow cytometry is increasingly recognized for diagnosing platelet disorders. Induction and accurate detection of phosphatidylserine exposure on the platelet surface requires proper recalcification of citrated blood samples, particularly in plasma from thrombocytopenic patients, in which residual citrate can limit free extracellular calcium availability.

**Objectives:**

To define optimal recalcification conditions for reliable measurement of procoagulant Collagen-And-Thrombin–activated (COAT) platelets in platelet-rich plasma (PRP) samples with low platelet counts.

**Methods:**

Fresh PRP from healthy donors was diluted with autologous platelet-poor plasma to simulate platelet counts from 160 × 10^9^/L to 15 × 10^9^/L. Undiluted PRP (control) and diluted samples were spiked with varying calcium concentrations and stimulated with convulxin and thrombin. Flow cytometry was used to measure annexin V and PAC-1 binding to assess procoagulant platelet generation.

**Results:**

For PRP with platelet counts ≥100 × 10^9^/L, no addition of calcium beyond the 2.5 mM already present in the buffer was required. For platelet counts between 30 and <100 × 10^9^/L, supplementation with 3 mM calcium restored procoagulant platelet generation to 85% to 115% of undiluted PRP control levels. Counts of 20 to <30 × 10^9^/L required 5 mM calcium to achieve an almost comparable restoration. Of note, higher calcium concentrations (>5 mM) impaired platelet function, highlighting the need to avoid excessive recalcification.

**Conclusions:**

Optimized PRP recalcification prevents underestimation of procoagulant platelet potential in thrombocytopenic samples. This practical approach addresses a key gap identified by the International Society on Thrombosis and Haemostasis Scientific and Standardization Committee and helps laboratories to ensure reliable platelet function testing in PRP samples from thrombocytopenic patients.

## Essentials

• Adequate extracellular calcium is required for accurate procoagulant Collagen-And-Thrombin–activated (COAT) platelet generation.
• Optimal recalcification for procoagulant COAT platelet formation was assessed by flow cytometry.
• Excessive remnant citrated plasma in low-count platelet-rich plasma samples binds calcium, causing false results.
• Proper recalcification prevents underestimating true procoagulant potential in thrombocytopenia.

## 1 Introduction

After dual stimulation with collagen (or convulxin [CVX], a collagen receptor glycoprotein VI agonist) and thrombin (THR), a distinct subpopulation of activated aggregatory platelets become procoagulant Collagen-And-Thrombin–activated (COAT) platelets [1,2]. This population is characterized by surface expression of phosphatidylserine (PS), retention of α-granule proadhesive and procoagulant proteins, and downregulation of the initially activated fibrinogen receptor glycoprotein IIb/IIIa in aggregatory platelets transitioning to a procoagulant phenotype [[3], [4], [5]]. It is also known that high and sustained free intracellular calcium concentration is a hallmark of procoagulant COAT, but not aggregatory platelets [1,6,7]. The influx of extracellular calcium via receptor/store-operated calcium entry pathways and sodium-calcium exchanger is critical for procoagulant phenotype development [1,8].

Platelet function tests generally employ blood drawn into tubes containing buffered sodium citrate as an anticoagulant. Flow cytometric detection of procoagulant platelets typically employs annexin V, requiring calcium to bind PS exposed on the platelet surface [9]. In our working protocol, platelet-rich plasma (PRP) is diluted with a calcium-containing buffer to a working platelet concentration of 5 × 10^9^/L, to have a standardized assay platelet count and optimal recalcification [1,10].

However, in case of severe thrombocytopenia, the lower the PRP dilution with calcium-containing buffer is required to reach a platelet concentration of 5 × 10^9^/L, the higher the amount of buffered citrated plasma remains in the assay. The increased citrate and the albumin concentration excessively chelate free calcium [11], whereas the lower pH reduces calcium entry into the platelet upon activation [12], which results in artefactually hampered procoagulant platelet formation and/or detection by annexin V binding [9,10].

A recent consensus report on platelet function testing by flow cytometry, issued by the Scientific and Standardization Committee of the International Society of Thrombosis and Haemostasis (ISTH), highlighted the critical role of recalcification in thrombocytopenic patient samples, stating that this aspect requires further studies [10].

Because accurate assessment of ionized free calcium is technically impossible in buffered citrated plasma, and since the latter inhibits procoagulant platelet formation in various ways, we aimed at determining empirically the optimal recalcification, using a functional assay, to assess the true procoagulant potential of thrombocytopenic samples.

## 2 Methods

### 2.1 Materials

Buffered citrate S-Monovettes tubes were obtained from Sarstedt (0.129 M buffered citrate pH 5.5). CVX was a kind gift from Prof K. J. Clemetson (Bern, Switzerland). Thrombin was purchased from Siemens. Cyanine 5 (Cy5)-annexin V and annexin V binding buffer (AVBB) containing calcium (working concentration, 140 mM NaCl, 2.5 mM CaCl_2_, 10 mM HEPES, pH 7.4) were obtained from Becton Dickinson. Phycoerythrin (PE)-conjugated PAC-1 (anti-human activated CD41/CD61 complex [PAC-1 epitope]) was purchased from Med Tech Trading (EXBIO Antibodies). Calcium chloride and calcium ionophore A23187 were obtained from Sigma Aldrich. The fibrin polymerization inhibitor Gly-Pro-Arg-Pro-OH (GPRP) was purchased from Bachem. Tyrode’s buffer (137 mM NaCl, 2.8 mM KCl, 12 mM NaHCO_3_, 1 mM MgCl_2_, 0.4 mM NaH_2_PO_4_, 10 mM HEPES, 3.5 g/L bovine serum albumin, 5.5 mM glucose, pH 7.4) and HEPES buffer (150 mM NaCl, 2.8 mM KCl, 1 mM MgCl_2_, 10 mM HEPES, pH 7.4) were produced in-house.

### 2.2 Blood collection and platelet preparation

This study was approved by the local Ethical Committee (CER-VD protocol 2018-00205) and conducted in accordance with the ethical standards of the Declaration of Helsinki. For the case studies, patients were enrolled at the outpatient clinic in the Service of Hematology of the CHUV. Donors had not ingested any medication influencing platelet function during the previous 10 days. Blood sampling, as well as PRP and platelet-poor plasma (PPP) preparation were performed as previously published [13].

### 2.3 Platelet kinetic assays

**Figure 1:**
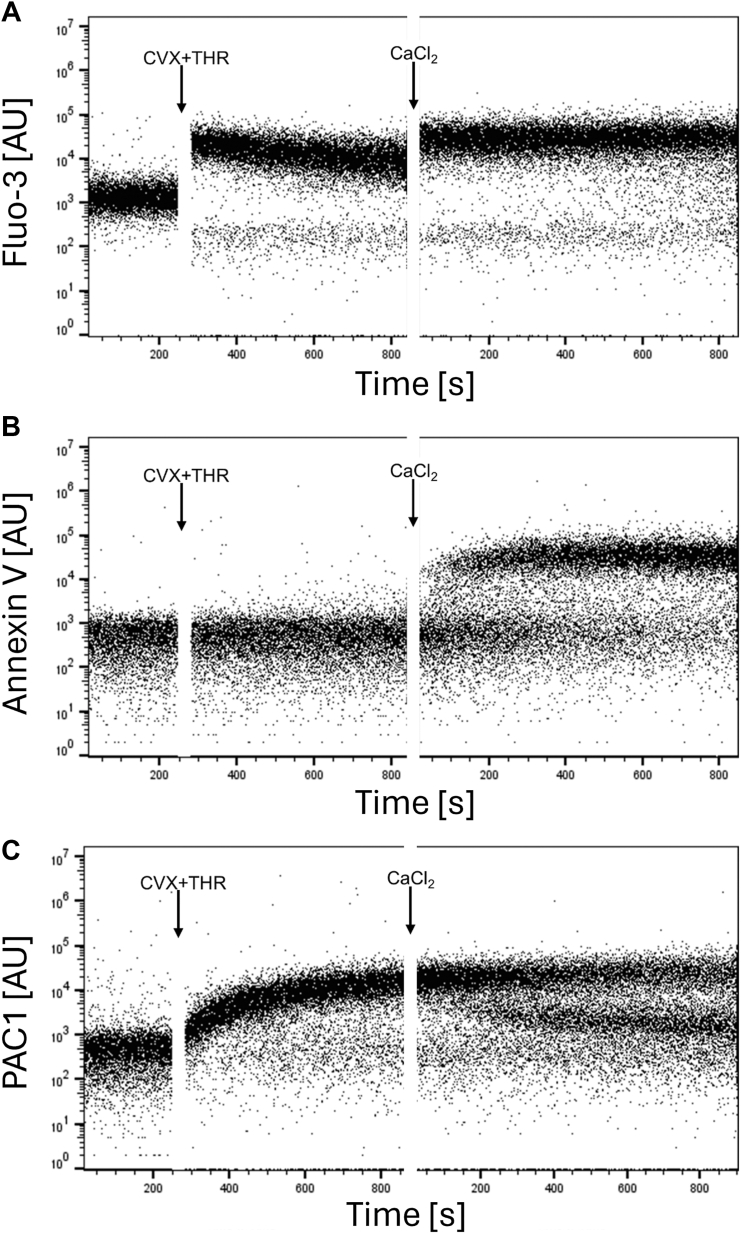
Extracellular calcium is required to sustain intracellular calcium elevation and promote procoagulant platelet formation. Platelet-rich plasma diluted in calcium-free Tyrode’s buffer was loaded with Fluo-3 AM (2 μM final concentration) and stained with Cy5-conjugated annexin V and PE-conjugated PAC-1 antibody. Flow cytometric kinetic acquisition was initiated with a 4-minute baseline, followed by stimulation with convulxin (CVX; final concentration 100 ng/mL) and thrombin (THR; final concentration 0.5 U/mL). After 10 minutes, 2 mM CaCl_2_ (final concentration) was added, and acquisition was resumed. (A) Intracellular calcium level monitored by Fluo-3 fluorescence, (B) phosphatidylserine exposure assessed by annexin V binding, and (C) fibrinogen receptor glycoprotein IIb/IIIa activation monitored by PAC-1 binding kinetics were measured over time. Data are representative of 3 independent experiments and are shown in arbitrary units (AU).

Kinetics experiments were performed as previously published [14,15], with the following modifications: the PRP was diluted in calcium-free Tyrode’s buffer. For intracellular calcium measurement, platelets were incubated with Fluo-3 AM (2 μM final concentration) for 1 hour at room temperature in the dark. After dye loading, platelets were costained with Cy5-conjugated annexin V and PE-conjugated PAC-1 antibody to assess PS exposure and fibrinogen receptor activation, respectively. Kinetic acquisition was performed by flow cytometry (BD Accuri C6, Becton Dickinson). Platelets were activated with addition of CVX (final concentration, 100 ng/mL) and THR (final concentration, 0.5 U/mL) (Figure 1). CaCl_2_ (2 mM final concentration) was added to assess calcium re-entry and procoagulant transformation.

### 2.4 Recalcification assay

Flow cytometry analysis was performed as previously published with slight modifications [16]. Briefly, Cy5-conjugated annexin V and PE-conjugated PAC-1 antibody were used. To simulate different levels of thrombocytopenia, PRP was kept undiluted (control, platelet count range = 473 to 254 × 10^9^/L) and diluted with autologous citrated PPP to obtain platelet counts of 160, 140, 120, 100, 80, 60, 40, 30, 20, and 15 × 10^9^/L. The different PRP preparations were further diluted to 10 × 10^9^/L with AVBB. To 50 μL of PRP (5 × 10^9^/L, final concentration stated) were added 26 μL of AVBB buffer and 2 μL of GPRP (2 mM). Then, 2 μL of calcium chloride were spiked, aiming at supplementing the following final calcium concentration: 0 (HEPES control), 1, 2, 3, 5, 7, 10, or 15 mM. The calcium-platelet preparations were gently mixed and incubated for 1 minute at room temperature. Then, 5 μL Cy5-annexin V and 5 μL PE-conjugated PAC-1 were added. Procoagulant COAT platelets were induced by combined stimulation with 10 μL CVX (100 ng/mL) and THR (0.5 U/mL) for 8 minutes at 37°C, as previously described [8,14]. Then, samples were diluted with AVBB to 0.5 × 10^9^ platelets/L, and 10,000 events were acquired in slow mode on the BD Accuri C6 flow cytometer. Platelets were gated according to their forward and side scatter properties. The recalcification was considered appropriate when the generation of procoagulant COAT platelets (PAC-1−/AnV+ events) was within the range of ±15% relative to the undiluted PRP control (without PPP dilution and calcium spiking). The range of ±15% was chosen according to the coefficient of variation observed for procoagulant COAT platelet generation.

### 2.5 Statistical analysis

Figures were generated using GraphPad Prism version 10.4.1 for Windows (GraphPad Software). Flow cytometry dot plots were generated using FlowJo version 10.9.0 for Windows (BD). Data are displayed as representative plots or mean ± standard error of mean (SEM) from separate experiments with platelets from different donors.

## 3 Results and Discussion

### 3.1 Extracellular calcium is required to promote procoagulant platelet formation

To investigate the necessity for extracellular calcium for procoagulant platelet generation, a kinetic flow cytometric acquisition was initiated using dual stimulation with CVX and THR. Intracellular calcium, annexin V and PAC-1 binding were continuously monitored. As shown in Figure 1A, CVX-plus-THR stimulation in the absence of extracellular calcium induced a rapid but transient increase in intracellular calcium, leading to PAC-1 positivity in all platelets (Figure 1B), consistent with an aggregatory phenotype. Of note, intracellular calcium levels subsequently declined over time, as expected [1,17]. Remarkably, when CaCl_2_ was added, it induced a sudden and sustained re-elevation of intracellular calcium, as already observed by others [7,18], accompanied by the appearance of annexin V binding (Figure 1C). Simultaneously, a dichotomous PAC-1 expression was observed, with a subset of platelets losing PAC-1 binding, consistent with the development of procoagulant COAT platelets. These findings are in line with previous studies demonstrating the critical role of extracellular calcium concentration in driving procoagulant platelet formation [1,19,20]. Our kinetic flow cytometry analysis provides additional mechanistic evidence, showing that sustained intracellular calcium elevation, PS exposure, and fibrinogen receptor inactivation occur after strong agonist stimulation only when sufficient extracellular calcium is available.

### 3.2 Recalcification restores procoagulant platelet generation in thrombocytopenic samples

**Figure 2:**
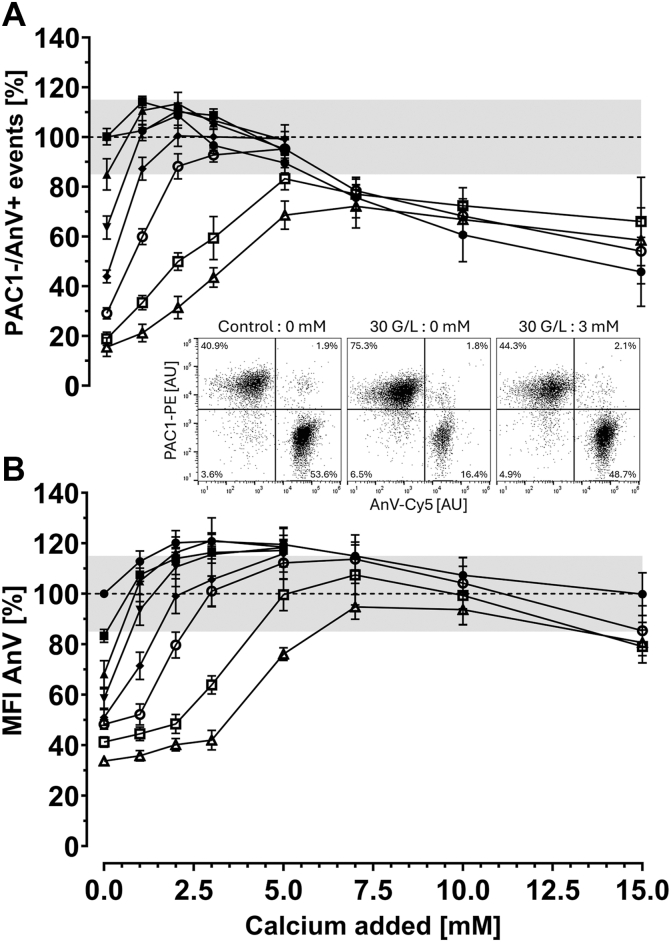
Procoagulant COAT platelet generation is impaired in samples with low platelet counts and is corrected with recalcification. Platelet-rich plasma (PRP) from healthy donors was kept undiluted (control) or diluted with autologous platelet-poor plasma to achieve final platelet concentrations ranging from 160 to 15 × 10^9^/L, simulating different levels of thrombocytopenia. Of note, the platelet count in the control ranged from 473 to 254 × 10^9^/L in PRP, and absolute procoagulant potential ranged from 30.6% to 53.3% PAC-1−/AnV+ events (*n* = 6). The platelet preparations were then diluted to 5 × 10^9^/L with calcium-containing AnV binding buffer. Subsequently, different concentrations of calcium were added (0 [HEPES buffer only], 1, 2, 3, 5, 7, 10, and 15 mM), and a double staining with PE-conjugated PAC-1 and Cy5-conjugated AnV was performed. Procoagulant COAT platelet generation was induced by combined stimulation with CVX (final 100 ng/mL) and THR (final 0.5 U/mL) for 8 minutes at 37 °C. (A) Generation of PAC-1−/AnV+ events (corresponding to procoagulant COAT platelets) and (B) Median fluorescence intensity (MFI) of AnV expression relative to the control. The gray shaded area represents the relative variation of 15% from the control as expected from the measurement of procoagulant COAT platelet generation. (Inset) Representative dot plots of procoagulant COAT platelet (PAC-1−/AnV+ events) generated in (left) control sample, (middle) PRP simulated at 30 × 10^9^/L without calcium supplementation, and (right) PRP simulated at 30 × 10^9^/L supplemented with 3 mM calcium. Of note, addition of extracellular calcium to the 30 × 10^9^/L simulated PRP restores both procoagulant COAT platelet generation and the loss of AnV MFI compared to control. Figure legend:  Control (473-254 × 10^9^/L),  160-100 × 10^9^/L,  80 × 10^9^/L,  60 × 10^9^/L,  40 × 10^9^/L,  30 × 10^9^/L,  20 × 10^9^/L,  15 × 10^9^/L platelet count in PRP. Data are shown as mean ± SEM (*n* = 3-6). AnV, annexin V; COAT, Collagen-And-Thrombin–activated; CVX, convulxin; Cy5, cyanine 5; G/L, 10^9^/L; PE, phycoerythrin; THR, thrombin.

We simulated different levels of thrombocytopenia by diluting PRP from healthy donors with autologous PPP to achieve final platelet concentrations ranging from 160 to 15 × 10^9^/L. As shown in Figure 2, for platelet counts in PRP ≥100 × 10^9^/L, no additional calcium supplementation beyond the 2.5 mM calcium already present in the standard buffer (AVBB) was necessary to preserve procoagulant platelet generation compared to controls (Figure 2A). For platelet counts in PRP between 30 and 80 × 10^9^/L, supplementation of 3 mM calcium (spiking 2 μL of 150 mM calcium chloride stock solution in a final volume of 100 μL) was sufficient to restore procoagulant platelet generation to levels within 85% to 115% of the control value. However, at a platelet count of 20 × 10^9^/L, supplementation of 5 mM of calcium (2 μL of 250 mM calcium chloride stock solution) was necessary to achieve an almost comparable restoration. Technical limitations were encountered for PRP with platelet counts <20 × 10^9^/L. In fact, at platelet concentrations ≤20 × 10^9^/L, recalcification did not fully restore procoagulant platelet generation within the target range of ±15% relative to control. Moreover, at platelet counts <15 × 10^9^/L, fibrin clot formation occurred in PRP due to high residual fibrinogen concentration and its activation by THR, despite increasing the concentration of GPRP to inhibit fibrin polymerization, precluding reliable flow cytometric analysis. However, assessing platelet function defects in patients with platelet counts <15 × 10^9^/L is unlikely to be clinically relevant, as the bleeding phenotype in such cases should be primarily attributable to the severe thrombocytopenia rather than an underlying functional defect. We noticed that calcium spiking corrected annexin V median fluorescence intensity more readily than COAT platelet generation, suggesting that the amount of extracellular calcium required for detecting procoagulant COAT platelets through annexin V binding is less limiting than the amount of extracellular calcium necessary to generate COAT platelets.

### 3.3 High extracellular calcium concentrations impair procoagulant platelet generation

An intriguing observation was the decreased COAT platelet generation when calcium concentrations >5 mM were added (Figure 2A), even when high residual citrated plasma remained (eg, platelet counts of 20 × 10^9^/L). Of note, annexin V binding was unaffected by high extracellular calcium concentration (Figure 2B).

While extracellular calcium is essential to trigger the sequences of events leading to the development of procoagulant platelet characteristics [21,22], several authors have demonstrated that supraphysiological extracellular calcium levels can impact integrin detection in resting platelets as well as platelet activation [23,24]. It is therefore possible that excessive calcium can lead to altered receptor activity and/or uncontrolled calcium fluxes impairing platelet functions [[25], [26], [27], [28], [29]].

### 3.4 Validation with samples from patients with thrombocytopenia and potential procoagulant defect

**Figure 3:**
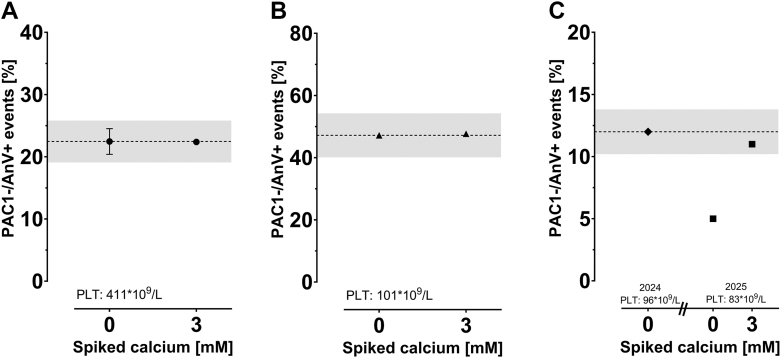
Recalcification is feasible with samples from patients with thrombocytopenia and procoagulant potential defect. Recalcification was used on 3 case studies to further demonstrate the performance of the recalcification algorithm. (A) The donor had a low procoagulant potential (but platelet counts within the reference range), and recalcification (3 mM calcium spiked) of platelet-rich plasma (PRP) did not induce an overestimation of the potential to generate COAT platelets. (B) The donor showed a low to normal platelet count with procoagulant potential within the reference range (25%-60%), and recalcification did not overestimate the potential to generate COAT platelets. (C) A donor with low platelet counts in PRP, previously found to have a low procoagulant potential (2024), was reinvestigated with recalcification a year later (2025) with a slightly lower platelet count in PRP. Recalcification confirmed a true procoagulant defect. The gray shaded area represents a relative variation of 15% from the unspiked sample, as expected from the measurement of procoagulant COAT platelet generation in the control sample. For each case study, the platelet count in the PRP is indicated on the bottom left. Only one replicate could be performed, except for (A), for which the baseline procoagulant potential was measured twice in independent experiments performed on separate days (mean ± SEM). AnV, annexin V; COAT, Collagen-And-Thrombin–activated; PLT, platelet.

We report the performance of our recalcification workflow in 3 case studies. As shown in Figure 3A, the recalcification of a sample with a platelet count within the reference range (441 × 10^9^/L in PRP and 253 × 10^9^/L in whole blood) with low procoagulant potential did not overestimate the percentage of procoagulant platelets generated (23.9% vs 22.4% without and with recalcification, respectively). Figure 3B reports similar results for a donor with moderate thrombocytopenia (101 × 10^9^/L in PRP and 76 × 10^9^/L in whole blood) and a procoagulant potential within expected values (47.2% vs 47.7% with 3 mM calcium). Finally, we analyzed a sample from a patient who was previously tested for low procoagulant potential (11%) with severe thrombocytopenia (96 × 10^9^/L in PRP and 47 × 10^9^/L in whole blood). When applying the recalcification workflow on the sample of the same patient 1 year later, showing a lower platelet count of 80 × 10^9^/L in PRP (55 × 10^9^/L in whole blood), we indeed highlighted a true procoagulant defect (5% vs 12% with 3 mM spiking; Figure 3C). These case studies further validate the feasibility and appropriateness of recalcifying samples to assess the true procoagulant potential.

## 4 Conclusions

Recent consensus guidelines from the ISTH Scientific and Standardization Committee have highlighted the challenges of accurate recalcification in thrombocytopenic samples for functional flow cytometry analysis, emphasizing the need for additional studies to address this methodological gap [10]. Our work addresses this unmet need. Here, we demonstrate that proper recalcification is a critical preanalytical step for accurate flow cytometric assessment of procoagulant platelet generation when working with PRP in thrombocytopenic conditions. Recalcification of PRP samples with a platelet count >100 × 10^9^/L is not required. In our hands, recalcification for platelet counts between <100 and 30 × 10^9^/L by spiking 3 mM calcium is feasible and necessary, whereas recalcification for samples between <30 and 20 × 10^9^/L by spiking 5 mM calcium is sufficient to overcome the impairment of procoagulant COAT platelet generation and annexin V binding induced by residual citrated plasma.

Low procoagulant platelet activity has been associated with clinically significant bleeding diathesis [16,30]. Therefore, failure to appropriately recalcify thrombocytopenic samples may lead to underestimation of the real platelet procoagulant potential. This preanalytical artefact could misleadingly suggest a platelet functional defect in addition to thrombocytopenia, potentially resulting in misdiagnosis and inappropriate clinical management. We recommend each laboratory to establish and validate its own recalcification protocol according to their flow cytometry workflow and analytical conditions.

## References

[1] Alberio L., Ravanat C., Hechler B., Mangin P.H., Lanza F., Gachet C. (2017). Delayed-onset of procoagulant signalling revealed by kinetic analysis of COAT platelet formation. Thromb Haemost.

[2] Polgár J., Clemetson J.M., Kehrel B.E., Wiedemann M., Magnenat E.M., Wells T.N.C. (1997). Platelet activation and signal transduction by convulxin, a C-type lectin from *Crotalus durissus terrificus* (tropical rattlesnake) venom via the p62/GPVI collagen receptor. J Biol Chem.

[3] Dale G.L., Friese P., Batar P., Hamilton S.F., Reed G.L., Jackson K.W. (2002). Stimulated platelets use serotonin to enhance their retention of procoagulant proteins on the cell surface. Nature.

[4] Alberio L., Safa O., Clemetson K.J., Esmon C.T., Dale G.L. (2000). Surface expression and functional characterization of alpha-granule factor V in human platelets: effects of ionophore A23187, thrombin, collagen, and convulxin. Blood.

[5] Dale G.L. (2005). Coated-platelets: an emerging component of the procoagulant response. J Thromb Haemost.

[6] Arachiche A., Kerbiriou-Nabias D., Garcin I., Letellier T., Dachary-Prigent J. (2009). Rapid procoagulant phosphatidylserine exposure relies on high cytosolic calcium rather than on mitochondrial depolarization. Arterioscler Thromb Vasc Biol.

[7] Keuren J.F.W., Wielders S.J.H., Ulrichts H., Hackeng T., Heemskerk J.W.M., Deckmyn H. (2005). Synergistic effect of thrombin on collagen-induced platelet procoagulant activity is mediated through protease-activated receptor-1. Arterioscler Thromb Vasc Biol.

[8] Aliotta A., Bertaggia Calderara D., Zermatten M.G., Alberio L. (2021). Sodium-Calcium Exchanger reverse mode sustains dichotomous ion fluxes required for procoagulant COAT platelet formation. Thromb Haemost.

[9] Josefsson E.C., Ramström S., Thaler J., Lordkipanidzé M., COAGAPO study group (2023). Consensus report on markers to distinguish procoagulant platelets from apoptotic platelets: communication from the Scientific and Standardization Committee of the ISTH. J Thromb Haemost.

[10] Jourdi G., Ramström S., Sharma R., Bakchoul T., Lordkipanidzé M. (2023). FC-PFT in TP study group. Consensus report on flow cytometry for platelet function testing in thrombocytopenic patients: communication from the SSC of the ISTH. J Thromb Haemost.

[11] Baird G.S. (2011). Ionized calcium. Clin Chim Acta.

[12] Marumo M., Suehiro A., Kakishita E., Groschner K., Wakabayashi I. (2001). Extracellular pH affects platelet aggregation associated with modulation of store-operated Ca^2+^ entry. Thromb Res.

[13] Aliotta A., Bertaggia Calderara D., Zermatten M.G., Alberio L. (2021). High-dose epinephrine enhances platelet aggregation at the expense of procoagulant activity. Thromb Haemost.

[14] Aliotta A., Krüsi M., Bertaggia Calderara D., Zermatten M.G., Gomez F.J., Batista Mesquita Sauvage A.P. (2020). Characterization of procoagulant COAT platelets in patients with Glanzmann thrombasthenia. Int J Mol Sci.

[15] Aliotta A., Bertaggia Calderara D., Zermatten M.G., Marchetti M., Alberio L. (2021). Thrombocytopathies: not just aggregation defects-the clinical relevance of procoagulant platelets. J Clin Med.

[16] Daskalakis M., Colucci G., Keller P., Rochat S., Silzle T., Biasiutti F.D. (2014). Decreased generation of procoagulant platelets detected by flow cytometric analysis in patients with bleeding diathesis. Cytometry B Clin Cytom.

[17] Aliotta A., Alberio L. (2022). Another piece of knowledge in the puzzle of procoagulant COAT platelets. J Thromb Haemost.

[18] Nagy M., Mastenbroek T.G., Mattheij N.J.A., de Witt S., Clemetson K.J., Kirschner J. (2018). Variable impairment of platelet functions in patients with severe, genetically linked immune deficiencies. Haematologica.

[19] Heemskerk J.W., Sage S.O. (1994). Calcium signalling in platelets and other cells. Platelets.

[20] Abbasian N., Millington-Burgess S.L., Chabra S., Malcor J.D., Harper M.T. (2020). Supramaximal calcium signaling triggers procoagulant platelet formation. Blood Adv.

[21] Veuthey L., Aliotta A., Bertaggia Calderara D., Pereira Portela C., Alberio L. (2022). Mechanisms underlying dichotomous procoagulant COAT platelet generation—a conceptual review summarizing current knowledge. Int J Mol Sci.

[22] Millington-Burgess S.L., Harper M.T. (2021). Cytosolic and mitochondrial Ca^2+^ signaling in procoagulant platelets. Platelets.

[23] Morris K., Masri S., Schnoor B., Papa A.L. (2025). Calcium levels modulate platelet function, platelet-cancer cell interaction, and cancer cell invasion. Sci Rep.

[24] Hu H., Forslund M., Li N. (2005). Influence of extracellular calcium on single platelet activation as measured by whole blood flow cytometry. Thromb Res.

[25] Leytin V., Gyulkhandanyan A.V., Freedman J. (2019). Platelet apoptosis can be triggered bypassing the death receptors. Clin Appl Thromb Hemost.

[26] Jackson S.P., Schoenwaelder S.M. (2010). Procoagulant platelets: are they necrotic?. Blood.

[27] Mattson M.P., Chan S.L. (2003). Calcium orchestrates apoptosis. Nat Cell Biol.

[28] Rizzuto R., Pinton P., Ferrari D., Chami M., Szabadkai G., Magalhães P.J. (2003). Calcium and apoptosis: facts and hypotheses. Oncogene.

[29] Jeong S.Y., Seol D.W. (2008). The role of mitochondria in apoptosis. BMB Rep.

[30] Segot A., Adler M., Aliotta A., Matthey-Guirao E., Nagler M., Bertaggia Calderara D. (2022). Low COAT platelets are frequent in patients with bleeding disorders of unknown cause (BDUC) and can be enhanced by DDAVP. J Thromb Haemost.

